# A Systematic Review of Interventions to Support Health, Wellbeing, and Job Satisfaction for Emergency Call Handlers in the Ambulance, Police, and Fire Services

**DOI:** 10.1002/smi.70233

**Published:** 2026-10-07

**Authors:** Emily Louise Phillpotts, Rhian Meaden, James Byron‐Daniel, Sarah Voss

**Affiliations:** ^1^ University of the West of England Bristol UK

**Keywords:** emergency call handlers, mental health, physical health, staff retention

## Abstract

Emergency call handlers (ECH) in ambulance, police, and fire services experience high levels of occupational stress which can have adverse health consequences. To systematically review interventions to support the health, wellbeing and employment of ECH working in the emergency services. Following PRISMA 2020 guidelines, academic databases and grey literature were searched [Last search: 06/02/25]. Studies of any design assessing physical or mental health or employment outcomes were eligible. Screening and quality appraisal were conducted independently, with data extraction completed and cross checked by two reviewers. The JBI family of tools was used to assess quality and risk of bias. Twelve studies met the inclusion criteria and were narratively synthesised into four categories: psychoeducation and resilience training, residential recovery programme, organisational wellbeing initiatives, and multicomponent interventions. Sample sizes ranged from *n* = 9 to *n* = 4020. The majority of interventions identified aimed to improve mental health, while physical health and employment outcomes were less frequently assessed. There was significant heterogeneity in study design which limited comparability between the studies. Most studies included a mixed sample of ECH and first responders and the methodological quality of included studies was variable. Some studies employed rigorous designs (e.g., RCTs), whilst others relied on observational methods. The included studies also demonstrated organisational barriers to the delivery of interventions in these settings. There was limited evidence for the effectiveness of interventions for supporting health and wellbeing or reducing stress amongst ECH. There is a need for role‐specific interventions for ECH.

**Systematic Registration:**

This systematic review was registered on PROSPERO: CRD42025639271

## 1 Introduction

Emergency call handlers are responsible for answering emergency calls, providing guidance and instructions to callers and coordinating personnel and resources. The terminology used to describe ‘Emergency Call Handlers’ (ECH) varies internationally and within the UK, but similar roles exist within emergency care settings, under titles such as call‐takers, emergency medical dispatchers and public safety telecommunicators. Although the configuration of these roles vary between countries, the description provided relates to ECH working within the UK. In the UK, ECHs are emergency service workers (clinical and non‐clinical) whose roles are triaging calls, managing and coordinating emergency resources (GOV.UK 2024). ECH can organise a mental health assessment and facilitate an appropriate response either through dispatching a crisis team or signposting to local resources (BBC News 2024). In the UK, ECH in the ambulance service triage emergency calls using software such as NHS Pathways or Medical Priority Dispatch System (MPDS). They provide the initial first aid advice to the caller or patient using a scripted algorithm (Perera et al. 2023). ECH are under pressure to respond to the next 999 call and are heavily monitored and audited to comply with software licencing requirements. Their actions can have implications for patient outcomes, for example, through the provision of telephone cardiopulmonary resuscitation (Berdowski et al. 2009).

ECH in the ambulance, police, and fire services report high levels of occupational stress and burnout (Coxon et al. 2016) alongside an increasing demand on their skills and demanding patterns of work (Torquati et al. 2019). This can have adverse physical and mental health consequences (Galbraith et al. 2021). A quantitative survey study of 720 police ECH showed that high levels of occupational stress are often attributed to organisational factors such as high work demands, absence of peer support, and conflict within working relationships (Galbraith et al. 2021). ECH in the police service specifically have a critical role in ensuring that information is stored accurately and safely (Stafford 2018). This is a crucial part of the investigation process and missing information can delay an emergency police response (Stafford 2018). Overall, ECH report that organisational hierarchies, a lack of training, managerial support, long working hours, little time to debrief, and limited supervision negatively impact psychological wellbeing (Coxon et al. 2016; Powell et al. 2022; Willis et al. 2020).

Common reasons for leaving ECH roles include inadequate staffing, low pay, poor shift patterns, and feeling underappreciated, unsupported, and undervalued (Snell and Grimwood 2020). Similarly, the experience of poor psychological wellbeing (Sprigg et al. 2007), health (Golding et al. 2017), and burnout often contributed to an increased desire to leave (Załuski and Makara‐Studzińska 2022). Staff working in the police control room report that their wellbeing is adversely affected by demands and workplace stress which leads to high sickness rates (HMICFRS 2018). Ultimately, high occupational demands contributed to poor overall health, including an increased likelihood of substance use, weight gain, poor sleep alongside chronic health conditions including hypertension and diabetes (Perez et al. 2021). This group may also be sedentary for extended periods of time with little opportunity for exercise (Galbraith et al. 2021). ECH can be at increased risk of overweight or obese compared to the general population (Gamble et al. 1991; Tsismenakis et al. 2009), and also show significantly increased cortisol levels compared to matched controls (Weibel et al. 2003). Shift work can make it challenging for ECH to make healthy dietary choices, engage in physical activity and have high quality sleep (Kent et al. 2016). Evidence has linked shift working to an increased risk of breast cancer, gastrointestinal disorders, cardiovascular disorders and mental health problems (Harrington 2001; Kent et al. 2016) ultimately leading to frequent burnout (Wisetborisut et al. 2014). This research demonstrates the need for interventions which mitigate stress and improve both physical and mental health for ECH.

Previous reviews have focused on the experience of occupational stress for staff working in the emergency services (Golding et al. 2017), and the impact on their health (K. Adams et al. 2015; Smith et al. 2019). Nevertheless, possible solutions to prevent and mitigate against these stressors are yet to be explored (Smith et al. 2019). A recent mapping review investigated mental health, wellbeing, and support interventions for ambulance service staff working in the UK (Clark et al. 2021), and found that there is little research into interventions to manage occupational stress at either an organisational or individual level.

The current systematic review will build on this work (Clark et al. 2021; Smith et al. 2019) but also look at interventions to support the physical and mental health of ECH. In the UK, the 999 switchboards can connect to six emergency services: ambulance, fire, police, coast guard, search and rescue, and bomb disposal. In alignment with previous literature and policy this review will look at three emergency services (ambulance, police, and fire services) (Golding et al. 2017; GOV.UK 2017). This is to maintain consistency across international emergency response services with broadly comparable operational procedures and pressures whilst limiting heterogeneity within identified studies. It is recognised that ECH may work within other emergency response services, however, these populations were outside the scope of this review.

This review aims to identify interventions to improve the health, wellbeing and employment outcomes amongst ECH in the ambulance, police and fire services across different countries and to examine the effectiveness of these interventions.

### 1.1 Review Question

What interventions are available to improve health, wellbeing and employment outcomes (e.g., job satisfaction, staff retention) for ECH working in the ambulance, police, and fire services?

#### 1.1.1 Population

Emergency call‐handlers in the emergency services (i.e., ambulance, police, and fire service dispatchers and call handlers).

#### 1.1.2 Interventions

Interventions (e.g., mental health support programmes, organisational interventions, peer support) designed to support health, wellbeing, and employment outcomes (e.g., job satisfaction, staff retention) of the emergency call handler workforce.

#### 1.1.3 Comparison

No comparison group. This review will look at differences between intervention type (e.g., charity resources, organisational programmes).

#### 1.1.4 Outcomes

Health (e.g., physical and mental health outcomes), wellbeing (e.g., happiness), and employment outcomes (e.g., job satisfaction, staff retention).

## 2 Methods

### 2.1 Search

Records were identified by searching a selection of bibliographic databases and grey literature. Searches were conducted between January and February 2025 [Date of last search: 06/02/25]. This review follows the PRISMA 2020 Guidelines (Page et al. 2021) (Figure 1).

**FIGURE 1:**
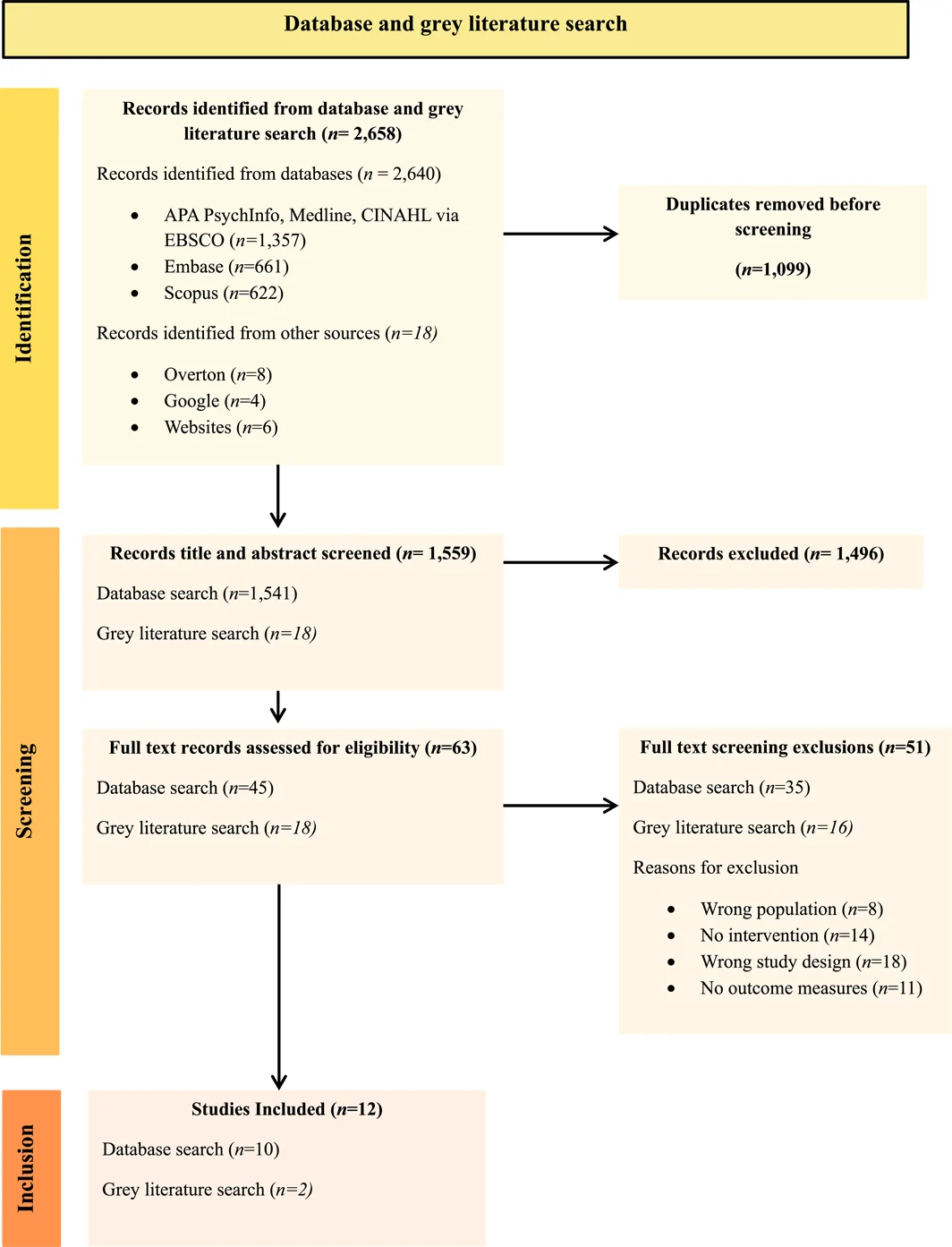
PRISMA diagram.

The following databases were searched:

- APA PsychInfo, CINAHL and MEDLINE via EBSCO
- EMBASE
- Scopus

Grey Literature Searches:

- Overton (first 50 results to capture the most relevant and recent results)
- Google (first 50 results to capture the most relevant and recent results)
- Relevant charity websites (e.g., Fire Fighters Charity, Mind, The Ambulance Staff Charity, Police Care UK)
- Relevant professional body websites (e.g., College of Paramedics, College of Policing, and National Fire Chiefs Council)
- Relevant service websites pages (e.g., staff support, and welfare webpages and staff benefit webpages)
- NHS Webpages

Grey literature search results were screened for initial relevance based on title and website summaries. Unrelated items were excluded at this stage, and only relevant sources were retained to be assessed against the full eligibility criteria (Figure 1).

A combination of terms was used to search for articles related to the concepts outlined in the review question (Table 1). The full search strategy can be found in Supporting Information S1: Appendix A.

**TABLE 1:** Overview of search terms used to define each concept.

Services	Emergency call handlers	Health	Wellbeing	Employment	Intervention
Ambulance	‘Emergency call handler’	‘Mental health’	Wellbeing	Job satisfaction	Initiative*
Police	Clinician*	‘Physical health’	Fatigue	Workforce	Support
Fire	Call taker*	Sickness	Stress	Staff retention	Resource*
	Dispatch*	Illness	Mood	Absenteeism	Programme*
	Call handler*	Exercise	Burnout		‘Peer support’
	999	Drinking	Exhaustion		Training
	911	Alcohol	Happiness		Strateg*
	‘Call centre’	Smoking			Guidance
	‘Dispatch officer’	Sleep			Intervention*
	‘Support staff’	Food			Implementation
		‘Healthy eating’			

*Denotes a truncation used within the search string to capture multiple word endings and plural forms.

### 2.2 Justification for Search Strategy

The terms used to describe emergency call handlers varies between countries. The search strategy was developed with an information specialist to capture relevant studies. It is possible that studies using alternative titles not included within the search strategy were not identified. The search strategy was developed in line with previous similar systematic reviews, including Lawn et al. (2020), with outcomes including psychological wellbeing, mental health, work related stress and physical health (Lawn et al. 2020). Specific outcomes within the search strategy were derived from synonyms of these concepts. The search strategy was derived to encompass broad conceptual terms for example, ‘physical health’ as suggested by previous reviews but also to include specific relevant terms as indicated by the literature for example ‘exercise’ (Galbraith et al. 2021). The search strategy was designed to capture formal and informal interventions aimed to reduce stress and promote physical and mental health outcomes for ECH. Informal interventions such as internal peer‐to‐peer support may be less likely to be reported in academic literature and thus, grey literature searches were also conducted (J. Adams et al. 2016).

No language restrictions were applied during the search and no changes were made to the review protocol after PROSPERO registration.

### 2.3 Eligibility Criteria

Eligibility criteria were broadly defined to encompass traditional interventions and also studies reporting on strategies to support wellbeing more generally, reflecting significant heterogeneity in the evidence in this field.

### 2.4 Inclusion Criteria

- Interventions where the target population included ECH working in the ambulance, police, and fire services (e.g., research interventions, local initiatives, and/or charity resources).
- Studies evaluating at least one intervention and its effect on at least one outcome measure relating to an aspect of physical or mental health, psychological wellbeing and/or an employment‐related outcome (e.g., job satisfaction or staff retention).
- Records published since 2000, to ensure the review reflected contemporary evidence about call handlers, whilst capturing as many applicable results to the study topic.
- Research conducted in countries in and outside of the UK.

#### 2.4.1 Exclusion Criteria

- Studies designed to support only frontline staff including paramedics, police officers, and fire fighters.
- Studies that did not evaluate the effects of at least one intervention on at least one outcome measure relating to an aspect of physical or mental health, psychological wellbeing and/or an employment‐related outcome (e.g., job satisfaction or staff retention).
- Existing reviews.

### 2.5 Resource Selection

References were managed using Zotero and all screening took place in Covidence. Articles identified were subject to title and abstract and full text screening by two independent reviewers (REDACTED) for inclusion. Reviewers were blind to each other's screening decisions at both the title and abstract and full text screening and met to resolve any disputes. Any disputes between the two reviewers would have been resolved through a nominated third reviewer. Data extraction was conducted independently by [REDACTED] and checked by [REDACTED] using a standardised extraction form, for all included records, including grey literature which was managed in MS Excel (Table 2, Supporting Information S3: Appendix B).

**TABLE 2:** Data extraction summary table (A blank copy of the full data extraction table can be found in Supporting Information S3: Appendix B).

Author, year, title, country	Service type	Design	Population type	Total sample size (number ECH)	Intervention or resource delivery mode, duration	Outcomes	Conclusion
Phung et al. (2022) The experiences and perceptions of wellbeing provision among English ambulance services staff: a multi‐method qualitative study UK (England)	Ambulance	Qualitative	ECH and frontline staff	*N* = 25 (*N* = not reported)	Formal organisation interventions	Mental health and wellbeing	Organisation level interventions are not accessed by all that need them and rely on self‐advocacy. Line manager role expansion is required to improve the provision of mental health and wellbeing support.
Anshel et al. (2013) Effect of a combined coping skills and wellness programme on perceived stress and physical energy among police emergency dispatchers: An exploratory study USA	Police	Action research	ECH	*N* = 9 (*N* = 9)	In‐person Weekly 10 Weeks	Stress, Job satisfaction, Body strength, Coping style for acute stress, Perceived physical energy	Increased use of avoidance coping, reduction in stress, more perceived physical energy. No changes in job satisfaction. Positive attitudes towards personal fitness coaching once a week.
Wild et al. (2020) Evaluating the effectiveness of a group‐based resilience intervention versus psychoeducation for emergency responders in england: A randomised controlled trial UK (england)	Police, fire, ambulance	Randomised controlled trial	ECH and frontline staff	*N* = 430 (*N* = not reported)	Resilience intervention in‐person/Psychoeducation intervention online Weekly 6 Weeks	Mental wellbeing, resilience, self‐efficacy, social participation, social support, depression, coping behaviours, rumination, anxiety, neuroticism	No significant differences between groups on any primary outcome measures at either post‐intervention or 3 month follow up. Resilience intervention was rated as more helpful than psychoeducation.
Weltman et al. (2014) Police department personnel stress resilience training: An institutional case study USA	Police	Quasi‐experimental	ECH and frontline staff	*N* = 14 (*N* = 2)	Stress resilience training app + optional 1 hour telephone mentoring for 4 weeks 6 Weeks	Emotional vitality Organisational stress Emotional stress Physical stress Heart rate variability	Improvements in emotional vitality (increased 25%), and physical stress (increased 24%). Participants valued the ability to apply the techniques to individual circumstances and situations.
(Marks et al. 2024) Improving mental health knowledge and reducing mental health stigma among public safety personnel: Comparison of live versus online psychoeducation training programs USA	Police, fire	Quasi‐experimental	ECH and frontline staff	*N* = 1686 (*N* = 43)	Training programme online versus in‐person. 1 hour	Mental health knowledge Mental health stigma	Brief psychoeducation training programme effectively increased knowledge of mental health and reduced mental health stigma. The training did not influence mental health self‐stigma. The mode of delivery (online vs. in‐person) did not make a difference on the effectiveness of the intervention.
Johnston et al. (2022) Perceptions and experiences of mental health support for ambulance trust employees UK (england)	Ambulance	Cross sectional study	ECH and frontline staff	*N* = 540 (*N* = 233)	Staying well service (Internal wellbeing service)	Work‐related stressful events Mental health sickness absence Service feedback form Acceptability of mandatory time to talk	Barriers to help‐seeking included stigma, fear and culture which prevents individuals from using internal support services. Women reported more positive perceptions of organisational support. Mandatory time to talk was acceptable to participants. Perception that colleagues would be less likely to seek support.
(Carleton et al. 2020) Mental health training, attitudes towards support, and screening positive for mental disorders Canada	Ambulance Police Fire	Survey	ECH and frontline staff	*N* = 4020 (*N* = not reported)	Formal support (healthcare professional provided) versus informal support (friends, colleagues, spouses)	Mental health screening Attitudes Mental disorder screening (PTSD, MDD, GAD, SAD, PD and AUD)	Mental health training was associated with increased willingness to access support and decreased odds of screening positive on any of the mental disorder screening tests. No specific category of training was superior at improving or protecting against poor mental health. There is variation in the willingness of public safety personnel to access both professional and non‐professional sources of support.
Hill et al. (2023) Mapping the health and wellbeing across the Firefighting career and assessing the current demands UK	Fire	Survey	ECH and frontline staff	*N* = 2224 (*N* = 90)	Employee assistance programs, peer support, social support, support for transitioning to retirement, personal development support, support for traumatic reactions	Mental wellbeing, Anxiety, work engagement, sources of occupational stress, intention to quit, burnout, impact of event scale, perceived social support, resilience coping, job satisfaction, satisfaction with life scale	Work engagement and job satisfaction were found to be predictors of wellbeing. Increased exercise had a statistically significant positive relationship with work engagement, job satisfaction, perceived social support, life satisfaction and resilient coping as well as a significant relationship with lower burnout and lower levels of anxiety, stress and depression.
Mind (2024) Blue light programme UK	Police, fire, ambulance, search and rescue	Qualitative	ECH	*N* = 312	Managing mental health in emergency services training Peer support scheme Call handler briefings Workplace wellbeing webinars Information booklets	Interviews, Site visits and observations to assess organisational attitudes towards mental health and wellbeing,	97% of individuals who attended the briefings said that they would ‘absolutely’ (85%) or ‘probably’ (13%) recommend the course. Some staff were unable to attend the training in their own time despite compensation. 87% agreed or strongly agreed that they knew more about different mental health problems after attending the briefings. 100% agreed or strongly agreed that they know where they can access support or advice regarding their own mental health after the course.
(Boothroyd et al. 2019) Evaluation of operation restore: A brief intervention for first responders exposed to traumatic events USA	Police, fire, ambulance	Mixed methods evaluation	ECH and frontline staff	*N* = 207 (*N* = 8)	Operation restore—residential programme 5 day programme and 90 day follow up	Post‐traumatic growth	91.9% of the participants experienced some degree of positive increase in their traumatic growth scores from pre to post intervention. The majority of improvements in post‐traumatic growth remained at 90 day follow up with the exception of personal strength.
Kamena and Galvez (2020) Intensive residential treatment programme: Efficacy for emergency Responder's critical incident stress USA	Police, fire, ambulance	Quasi‐experimental study	ECH and frontline staff	*N* = 142 (*N* = 6)	West coast post trauma retreat (WCPR) 6 days	Symptom assessment for emergency responders (SAFER)	WCPR contributed to lowering participants post‐traumatic stress symptoms and overall symptoms of stress, anger, reactivity, generalised anxiety, obsessive‐compulsive symptoms, panic attacks, somatic symptoms and suicidal thoughts.
Cantrell (2011) The change in first responders trauma symptoms after participation in a residential recovery programme USA	Police, fire, ambulance	Quasi‐experimental	ECH and frontline staff	*N* = 147 (*N* = 5)	West coast post trauma retreat 6 days	Trauma symptom inventory, Post traumatic stress	Symptoms were significantly reduced on all ten scales of the trauma symptom inventory; anxious arousal, depression and defensive avoidance showed the most improvement. No association between the TSI summary scale and either primary or secondary trauma.

### 2.6 Study Quality Assessment

Published literature was assessed independently by two reviewers (REDACTED) using the JBI family of critical appraisal tools to assess risk of bias (Lizarondo et al. 2024) (Supporting Information S4: Appendix C). This included risk of bias checklists for methodologies including analytical cross sectional studies, case control studies and cohort studies (Moola et al. 2020, 7), qualitative research (Lockwood et al. 2015), quasi‐experimental studies (Barker et al. 2024) and randomised controlled trials (Barker et al. 2023). Grey literature was evaluated and assessed according to the AACODS checklist and appraised based on: Authority, Accuracy, Coverage, Objectivity, Date, and Significance (Tyndall 2010).

## 3 Results

### 3.1 Overview of Included Studies

A total of *n =* 12 studies were included in this systematic review. *N* = 10 studies contained a combined sample of ECH and frontline staff and *n =* 2 studies contained an ECH‐only sample. Studies were conducted in the United Kingdom, Canada or the United States. Populations included the ambulance, police and fire service workforce with frontline roles and ECH in these services. Study designs included qualitative studies, quasi‐experimental studies, cross sectional surveys, mixed methods evaluations and one randomised controlled trial. Sample sizes ranged from *n* = 9 (Anshel et al. 2013) to *n = 4*020 (Carleton et al. 2020) with varying representation of ECH within the total sample (Table 2). Outcome measures assessed mental health (including anxiety, depression, post‐traumatic stress, resilience) and measures of health, wellbeing, stigma and job satisfaction. Where ECH specific findings were reported, this was descriptive and not related to ECH experiences of intervention or an outcome measure within the scope of this review. Interventions were delivered in person and/or online and the duration ranged from one‐off interventions to 6‐day residential programmes. There was significant heterogeneity in study design and outcome measures of included studies and as such a narrative synthesis was chosen as the appropriate analysis method.

The results were organised into four categories reflecting the primary focus of each intervention: psychoeducation, resilience and mental health training, residential recovery programmes, organisational wellbeing and perception of support and multi‐component interventions. For each intervention type, the studies are synthesised to address the main aims including; intervention characteristics and a summary of evidence for the effectiveness of intervention.

### 3.2 Psychoeducation, Resilience and Mental Health Training

Psychoeducation interventions aimed to improve mental health knowledge, reduce stigma and promote help‐seeking behaviours. These interventions involved improving knowledge of mental health, coping strategies and support services (Marks et al. 2024). Resilience based interventions applied stress management techniques to improve coping skills and self‐management of stress.

Marks et al. (2024) delivered an in‐person and online version of a psychoeducation training session, looking at job‐related stressors, how stress impacts physical, cognitive and emotional functioning, followed by information about managing PTSD, depression, anxiety, anger, substance misuse and sleep problems (Marks et al. 2024). Wild et al. (2020) compared the effectiveness of group‐based resilience training to a psychoeducation intervention (Wild et al. 2020). The resilience intervention comprised a group‐based course where participants attended a 2.5‐h session once a week for 6 weeks with homework exercises in between. The psychoeducation group completed online modules on sleep, stress, depression, anger, mindfulness and post‐traumatic stress disorder. Weltman et al. (2014) conducted an evaluation of a 6‐week intervention using the Stress Resilience Training System (SRTS) app. The app includes information on the physiology of stress and resilience, self‐regulation techniques, and a series of games designed to increase resilience. Participants also received a one‐hour mentoring session each week for the first 4 weeks. Finally, Anshel et al. (2013) evaluated a 10‐week intervention to build resilience by combining wellness coaching (exercise and nutrition) with coping skills training. The intervention was delivered over the course of three two‐hour seminars to fit around dispatcher shifts.

The studies included within this category compared both in‐person and online psychoeducation interventions. Both modalities of intervention delivery improved mental health knowledge and reduced stigma (Marks et al. 2024). However, the study lacks reliability as the in‐person session included discussion while the online session was video only, which may have influenced the experience of the intervention and could influence the reliability of the comparison between groups. Additionally, there was an imbalance in the allocation of participants to the different groups such that *n* = 1570 attended the online training and *n* = 116 attended the in‐person training. The sample also had a high attrition rate with *n* = 871 (55%) in the online group and *n* = 44 (38%) in the in person group completing the pre and post questionnaires (Marks et al. 2024). The large differences between groups and variation in intervention delivery confounds the conclusions drawn. By contrast, a randomised controlled trial comparing resilience training with an active psychoeducation intervention found no significant differences between the two on any of the outcome measures including, resilience, wellbeing, self‐efficacy, social participation, social support (home), social support (work), days off work/week, and confidence to manage mental health at post‐intervention (Wild et al. 2020). Together with the findings from Marks et al. (2024) this could indicate that whilst psychoeducation and resilience interventions may improve knowledge and stigma they may not improve health outcomes.

In the Weltman et al. (2014) study, the use of the app was correlated with improvements in emotional vitality and physical stress. However, the results of this intervention should be interpreted with caution due to the small sample size of *n =* 14, with only *n =* 2 ECH (Weltman et al. 2014). Anshel et al. (2013) also had a small sample size (*n* = 9) and did not include a control group however, showed an improvement in dispatchers lower and upper body strength scores following the fitness part of the intervention. Anshel et al. (2013) was the only included psychoeducation study with a sample of only ECH, which adds strength to this study in comparison to Marks et al. (2024), Wild et al. (2020) and Weltman et al. (2014). There is mixed evidence for the effectiveness of psychoeducation, resilience and mental health training interventions. Whilst Anshel et al. (2013) and Weltman et al. (2014) provide evidence that resilience interventions improve stress, energy and strength, the sample sizes are small and neither study recruited to a control group. However, Wild et al. (2020) had a stronger methodological design, larger sample size and 3‐month follow up but reported no significant differences between the two intervention arms (resilience training vs. psychoeducation). This suggests that psychoeducation, resilience and mental health training interventions may improve knowledge of symptoms of poor mental health and coping mechanisms, however the evidence for the effectiveness of these interventions on improving outcomes for ECH is weak.

### 3.3 Residential Recovery Programmes

These studies report residential programmes which targeted trauma symptoms such as Post‐Traumatic Stress Disorder (PTSD). These multi‐component residential programmes involved multi‐day stays and aimed to target cumulative occupational stress.

Kamena and Galvez (2020) and Cantrell (2011) both assessed the West Coast Post Trauma Retreat (WCPR), a 6 day residential programme including psychoeducation, individual therapy, peer support and EMDR. Another residential retreat, ‘Operation Restore’, involved a 5‐day intervention with 90 day follow up based on the Critical Incident Stress Model (CISM) and the Critical Incident Stress Debriefing (CISD) model (Boothroyd et al. 2019). The retreat included Eye Movement Desensitisation and Reprocessing (EMDR) sessions, small group peer focused activities and educational presentations.

For the WCPR programme, participants showed a reduction in stress, anger, hostility, generalised anxiety, impairment, obsessive compulsive symptoms and suicidal thoughts (Kamena and Galvez 2020) and reduction across all ten scales in the TSI in anxious arousal, depression and defensive avoidance (Cantrell 2011). Following the retreat, participants reported an increase in post‐traumatic growth, particularly in appreciation for life and personal strength, which was maintained at a 90‐day follow up, the longest follow up of all included studies (Cantrell 2011; Kamena and Galvez 2020).

Nevertheless, Kamena and Galvez (2020) reported only *n =* 6 dispatchers in a total sample of *n =* 142 and Cantrell (2011) reports only *n =* 5 dispatchers in a total sample of *n =* 147. Therefore, whilst both Cantrell (2011) and Kamena and Galvez (2020) found improvements in participants' symptoms, the limited number of ECH participants, limits the extent to which the WCPR can be considered effective for ECH. Additionally, in Boothroyd et al. (2019) participants volunteered themselves to attend the retreat; this exposes the intervention to self‐selection bias, particularly as there was no control group and thus participants could be affected by the placebo effect of attending the residential retreat itself. Similarly, there were only 8 call handlers and dispatchers in the total sample (*n =* 207) in Boothroyd et al. (2019). All three residential retreats were conducted in the USA and are resource intensive, thus, the generalisability and practicality of residential interventions is uncertain.

All three studies also focused on mental health and stress related outcomes such as post traumatic growth but did not measure any other health outcomes. Overall, whilst multicomponent residential treatment programmes may cover a range of intervention types (psychoeducation, resilience training and coping strategies) most of these studies used single arm designs with no comparison group; one included a non‐randomised waiting list comparison (Kamena and Galvez 2020), which reduces but does not remove this limitation. Alongside self‐selection bias and the small proportion of ECH within the total sample limits the generalisability of these findings.

### 3.4 Organisational Wellbeing and Perception of Support

This review also included studies which looked at ECH perception of support including organisation level interventions for example internal wellbeing services. The included studies within this category predominantly evaluate acceptability rather than intervention effectiveness. Phung et al. (2022) conducted a documentary analysis and semi structured telephone interviews with wellbeing leads to assess wellbeing policy implementation in English ambulance service NHS Trusts. Johnston et al. (2022) conducted a survey of how ambulance staff perceived organisational support including attitudes towards seeking professional help, experience of the internal staying well service, and the acceptability of a mandatory time to talk session about work related stressors. Carleton et al. (2020) assessed the impact and attitudes towards different kinds of organisational support including mental health training, attitudes towards mental health support and mental disorder screening (depression, anxiety, post traumatic stress disorder (Johnston et al. 2022)r, social anxiety, panic disorder and alcohol use disorder).

The provision and effective implementation of organisation level interventions, for example internal staying well services, was strongly associated with organisational culture and manager attitudes towards help‐seeking (Johnston et al. 2022). Some participants suggested there was an increase in the willingness of personnel to seek help from health and wellbeing services whereas others felt that cultural stigma within teams contributed to presenteeism and disincentivised help‐seeking (Phung et al. 2022). Health and wellbeing leads suggested that geography was a challenge to intervention implementation such that teams are often located physically far apart (Phung et al. 2022). Therefore, the lack of both availability and accessibility of centralised organisation limited their usefulness in appropriately supporting ECH and ambulance personnel (Phung et al. 2022).

Johnston et al. (2022) found that whilst participants were aware of an internal ‘Staying Well Service’ and more than one third reported making contact for mental health support, there were mixed experiences of accessing organisation level interventions and the inconsistent implementation of these interventions negatively influenced help‐seeking behaviours. Carleton et al. (2020) found that there was a lack of clarity amongst staff about what interventions were available to them and how staff could access them. Whilst there was knowledge of the existence of professional sources of support and non‐professional sources, there was significant individual variation in who chose to seek help (Carleton et al. 2020). This section of the review identifies interventions but doesn't provide strong evidence for their effectiveness due to the descriptive and observational study design. Phung et al. (2022), Johnston et al. (2022) and Carleton et al. (2020) demonstrate that there is significant variation in the use and experiences of organisation level interventions however, further research is needed to test their efficacy.

### 3.5 Multi‐Component Approaches

Mind's Blue Light Programme (Mind 2024), included ECH from the police, fire, ambulance and search & rescue services. Mind created a programme of support including action plans, mental health awareness, resilience and workplace wellbeing resources. The other multi‐component intervention was the ‘Mapping Health and Wellbeing of Firefighters’ report (Hill et al. 2023) which provided an overview of a programme of work which included firefighters, dispatchers and call handlers and identified large variations in wellbeing between roles and seniority noting the specific challenges ECH face (*n =* 3084 total responses received, *n =* 2224 responses retained for analysis) (Hill et al. 2023). Unlike Mind's Blue Light Programme, this report took a top‐down approach emphasising the need for tailored interventions throughout different career stages and for different role types. However, the report did not link to any longer‐term outcomes.

In the Mind (2024) programme, 87% of participants across all services demonstrated an improvement in their knowledge of mental health and how to improve resilience (Mind 2024). Participants felt that the culture around mental health was changing which had a positive impact on staff, however, expressed concerns about the programme maintaining momentum. Additionally, no aspect of the programme looked at improving organisational attitudes (Mind 2024). Further, the ‘Mapping Health and Wellbeing of Firefighters’ report (Hill et al. 2023) indicated that length of service and limited amount of time to spend with close family and friends can influence experience of poor health due to lack of peer support. Additionally, the nature of shift‐work, led to poor physical activity, nutrition, poor posture, increased fatigue and increased experience of stress (Hill et al. 2023). These two reports together, indicate that whilst multi component interventions combine different elements which may support outcomes such as wellbeing, they rely on self‐report and observational measures. Therefore, the evidence of their effectiveness is limited. Additionally, these two pieces of work were carried out by two separate professional bodies and heterogeneity in outcome measures could be due to competing priorities, different objectives and different target populations.

Overall, methodological quality across all included studies was moderate to high with key limitations being lack of randomised control groups and limited comparability between participant groups in quasi‐experimental designs.

## 4 Discussion

This systematic review aimed to identify and evaluate interventions to support ECH working in the ambulance, police and fire services. Mental health outcomes were most frequently reported in all studies with several interventions reporting reductions in stress, anxiety and emotional exhaustion. Physical health outcomes included, musculoskeletal health issues (Phung et al. 2022), energy (Anshel et al. 2013) and alcohol consumption and exercise (Hill et al. 2023). Employment outcomes consisted of days off work per week (Wild et al. 2020), work engagement, intention to quit (Hill et al. 2023) and job satisfaction (Anshel et al. 2013). The underrepresentation of interventions to support physical health outcomes in comparison to mental health outcomes is recognised, indicating that current interventions largely focus on improving mental health.

Furthermore, the overall quality of the included studies was variable. Only one included study adopted a randomised controlled trial method, other study designs lacked methodological rigour relying on observational methods. Outcomes were often inconsistently reported and few studies included long term follow up. Considerable conceptual and methodological heterogeneity exists amongst the included studies which poses challenges for the narrative synthesis. Differences in design, intervention delivery and outcome measures limited the ability to draw comparisons across studies.

The uptake and impact of interventions identified within this review was inconsistent across settings. Insights from implementation science offer frameworks for understanding how organisational context (culture, leadership, engagement and geography) influences intervention implementation (Shelley and Rogers 2025). This systematic review demonstrates a gap between intervention design and delivery as indicated in Phung et al. (2022) and Johnston et al. (2022) where barriers such as stigma, inconsistent provision, access, and lack of managerial support limited the implementation of interventions. Additionally, intervention fidelity and mechanisms of change were not identified nor measured in the included studies which makes it challenging to determine what works, what doesn't work and why within the ECH workforce.

Interventions included in this review often relied on participants who had volunteered their own time to participate. This introduces a self‐selection bias which limits the external validity of the study as the sample is not generalisable to the wider population. For example, Weltman et al. (2014) offered an additional mentoring session, which some found helpful, however, this wasn't able to be accessed by all participants in the study. Thus, those who were unable to spare the time to take part in additional mentoring didn't receive the benefit. Future interventions should aim to be delivered in paid time to ensure they are equally accessible to staff. Some participants also felt that interventions were repetitive of training they had already received, or that they didn't have experience of poor mental health and thus the content wasn't applicable to them (Mind 2024). For organisation level interventions, it is possible staff may have been aware of Staying Well Services or Employee Assistance Programmes, but these generally had poor uptake, which was compounded by inconsistent implementation. Therefore, there is limited evidence from a small number of heterogenous studies with a number of confounding variables that current interventions to support ECH are effective.

The studies included within this review included evidence from both the UK, USA and Canada. However, the policy implications are considered in relation to UK emergency services, given its relevance within this context. For example, the NHS Long Term Workforce Plan (2023) highlights staff retention as a key priority area (NHS England 2023). Moreover, the National Partnership Agreement: Right Care, Right Person (RCRP) emphasises the need for a timely and appropriate response from the emergency services (GOV.UK 2024). This places pressure on ECH to make complex clinical decisions under time constraints. As such, the College of Policing (College of Policing 2025) and National Fire Chiefs Council (NFCC 2025) both emphasise the need for improved wellbeing support for ECH. Yet, there is limited evidence from this review of the acceptability, practicability, effectiveness, affordability, spillover effects and equity (APEASE criteria, Michie et al. (2014)) of these interventions to reduce stress and improve health outcomes of ECH.

ECH experience an increased likelihood of substance use, weight gain, and poor sleep alongside the development of chronic health conditions including hypertension and diabetes (Perez et al. 2021). Compared to administrative staff and frontline firefighters, ECH were twice as likely to engage in habitual smoking behaviours and experience greater sleep disturbance (Turner et al. 2018). Dispatchers were found to have higher resting heart rates, increased incidence of cardiovascular disease, and musculoskeletal issues which has consequences for the experience of work‐related disability (Oldenburg et al. 2014). The lack of role specific interventions identified in this review for ECH, suggests a disconnect between policy aims and the evidence base. Emerging research continues to investigate how organisational environments predict staff turnover and retention (Alkan et al. 2024) which reinforces the need for role specific interventions for ECH.

There are limitations within this review including the sensitivity and specificity of the search strategy and database selection. Five studies were based in the UK and six were based in the USA and one in Canada, possibly due to the variation in terms used to define the role of ECH. The authors acknowledge this as a limitation within the search strategy. Studies conducted within the UK were also based within England and do not represent ECH in the other nations. It is possible that the search conducted did not capture all relevant studies given the variation in terms used to describe ‘emergency call handlers’ internationally. Whilst the scope of the review was international, evidence was limited to studies conducted in the UK, USA and Canada, limiting the generalisability of the findings beyond the context of these services. Additionally, there were challenges with data extraction and synthesis given the significant heterogeneity in identified records. Of the included studies, only 2 were conducted with a sample of ECH only, whereas most studies included a mixed sample of ECH and frontline personnel and did not disaggregate the findings by job type to examine intervention effectiveness. Descriptive statistics showed that ECH often formed a very small proportion of the total sample. This was also revealed in the quality appraisal which showed common methodological limitations such as lack of control groups, small sample sizes and limited follow up assessment. (Supporting Information S4: Appendix C). Consequently, while some studies reported improvements across outcome measures, confidence in these findings is limited by methodological weaknesses. Therefore, there is insufficient robust evidence for the effectiveness of interventions to support ECH specifically.

This systematic review indicates that top‐down, organisation‐level interventions such as line manager training as in Phung et al. (2022) are needed to overcome institutional barriers for call handlers accessing support. Where interventions (e.g., Anshel et al. (2013)) included individual support such as mentoring, participants found this beneficial and individual‐level interventions for ECH are an important consideration for future research to explore. Future research may also consider the following:

- Include emergency service personnel from other emergency services including coast guard, search and rescue, and bomb disposal.
- Role‐specific interventions for ECH at an organisational and individual level.
- Use of behaviour change theory to design interventions that target specific health behaviour outcomes.
- Interventions which assess long term outcomes for example stress reduction and impact on health over time.
- Intervention evaluation including identifying mechanisms of change and intervention fidelity.
- Interventions which focus on physical health outcomes, stress reduction, wellbeing and employment outcomes.

## 5 Conclusion

Emergency Call Handlers (ECH) experience significant organisational stress which has adverse consequences for their mental health, physical health and job satisfaction. This review identifies a limited and heterogeneous evidence base of interventions to support staff working in the ambulance, police and fire services. Studies mostly included a combined sample of ECH and frontline staff therefore, the ability to draw conclusions about these interventions for ECH specifically is limited. Additionally, all studies were either based in the UK, USA or Canada limiting the generalisability of this systematic review. The risk of bias in study methodologies varied including one randomised controlled trial, however, other records relied on weaker designs including quasi‐experimental and observational methods. Therefore, the evidence supporting the impact of these interventions on stress, health, wellbeing and employment outcomes is weak. This review also identified barriers to centralised intervention delivery, given that ECH are often geographically located far apart, making intervention delivery challenging. This review offers suggestions for further research to develop interventions that are visible, accessible, feasible and acceptable. Future research should focus on role‐specific interventions at an organisational and individual level for ECH, that assess the long‐term impact on stress, physical and mental health, wellbeing and employment outcomes.

## Ethics Statement

This study was a systematic review of previously published literature, and no new primary data was collected from human participants. Thus, formal ethical approval was not required.

## Conflicts of Interest

The authors declare no conflicts of interest.

## Supporting information


Supporting Information S1

Supporting Information S2

Supporting Information S3

Supporting Information S4

## Data Availability

The search strategy, data extraction form and data quality assessment output are available. The review protocol was prepared but not published and can be accessed upon request to the corresponding author. Any additional data or materials used in this review can be made available upon request. This review followed the PRISMA 2020 guidelines for transparency and reproducibility.
