# Supplementary material for: A Systematic Review of Interventions to Support Health, Wellbeing, and Job Satisfaction for Emergency Call Handlers in the Ambulance, Police, and Fire Services
Source: Stress Health. 2026 Oct 7;42(5):e70233. doi: 10.1002/smi.70233 (PMC13646713; doi:10.1002/smi.70233)
Supplement: Supplementary file 1 [file SMI-42-e70233-s002.pdf]

Record No.	Source	Date of search	Date of record	Organisation Name	Subject	URL	Service (ambulance, police, fire)	Intervention / Initative / Resource	Outcomes	Screening (EP)	Screening (RM)
1	Overton	2/7/2025	1/29/2019	London Ambulance Service NHS Trust	Meeting minutes of the London Ambulance Service NHS	Record 1 2019-01-29-Trust-Board-	Ambulance	1) Continue to benchmark lost time / sickness records 2) Support with the development of policies and strategies to manage stress. 3) Ensure fatigue is considered in operational risk assessments. 4) Support driving standards regarding any programmes for driver fitness.	Collect and analyse sickness data with respect to health and safety. No other specific outcomes reported.	No. - No intervention	No - not intervention based
2	Overton	2/7/2025	11/26/2019	London Ambulance Service NHS Trust	Meeting minutes of the London Ambulance Service NHS	Record 2 2019-11-26-Trust-Board-	Ambulance	1) Employee relations case management system and occupational health interface project will support managers to monitor sickness rates. 2) Full review of occupational health services focusing on immunisation programmes and physiotherapy. 3) Clinical education strategy workshop	Updake of services & staff sickness rates trust wide	No - No intervention	No - not intervention based
3	Overton	2/7/2025	Sep-22	The Inspector-General for Emergency Management authored and published by the Victorian Government, 1 Treasury Place, Melbourne	Review of Victoria's emergency ambulance call answer performance	Record 3 Victoria, Australia Emerg	Ambulance, Fire, Police	1) face to face counselling with employees, 2) psychological education and self care sessions 3) various seminars on topics including resilience, energy management, and managing vicarious trauma, 4) support for the leadership teams, 5) general walk arounds and welfare checks.	1) Use of welfare absence and welfare breaks 2) People Matters Survey (standardised survey to assess workplace culture) 3) Implementation of shift restricitions for 12 hour shifts.	No - outcomes focused on performance not health, wellbeing or job satisfaction	No - performance metrics focus
4	Overton	2/7/2025	6/14/2022	Toronto Police Service	Better support for staff, improved information management and outcomes	Record 4 Toronto Police Service	Police Service	1) Making technological imrpovements (an automated system to streamline the call taking process, e.g. Criteria based dispatching program (similar to MPDS). 2) Opportunities to update or improve clarity in call taker manual (for certain event types) 3) Training opportunities for communications operators.	None specified	No - outcomes focused on performance not health, wellbeing or job satisfaction	No - performance/service metrics focus
5	Overton	2/7/2025	Jun-23	North Western Ambulance Service Annual Repor	People Plan (p.47)	Record 5 Complete-NWAS-Ann	Ambulance	People Plan (2018) - reviewed annually. Reflects three themes: Develop, engage and empower and six priorities: 1) recruitment and retention, 2) developing potential, 3)wellbeing, 4) Inclusion, 5) Leadership, 6) Innovation and improvement. Key Health and wellbeing interventions: Health and wellbeing leads, consultant psychologist, work and wellbeing passport, mental health continuum delivery group, pro-active, confidential and emotional wellbeing support telephone calls	National Staff Survey Result (2022) National Quarterly Pulse Sur	No - outcomes focused on performance not health, wellbeing or job satisfaction	No - performance metrics focus
6	Overton	2/7/2025	2017/18	East of England Ambulance Service	Annual Report 2017/18	Record 6 East_of_England_Amb	Ambulance	Citing high turnover rates for call handler staff. To support this, built in resilience into recruitment screening and emergency call hander training. Retention strategies have focused on wellbeing interventions to support the mental health of the workforce. Included health and wellbeing seminars on issues including suicide prevention and launched a wellbeing bus that travels across the region to support staff with any physical, emotional, social, and psychological issues.	Retention rates / Staff Survey	No - didn't report on outcomes	No - intervention outcomes not reported
7	Overton	2/7/2025	9/4/2020	Health Information and Quality Authority	Review of guidance for the operation of emergency med	Record 7 Guidance-for-emerge	Ambulance	Guidance for workforce health and wellbeing included: (1) watch for atypial signs in staff behaviours and address as soon as possible, (2) communicate with staff regarding new measures and provide information regarding avaliable supports (eg. Peer support, employee assistance programme, employee wellness programme, critical incident stress management support), (3) develop formal/informal wellnes plans, (4) encourage EMS personnell to create structure at home to help create certainty and normalise the home, (4) ensure staff take respective rest periods including active breaks, (5) ensure staff have designated spaces for food and rest, (6) restrict visitation to facilitites	None specified	No - guidance document not intervention	No - not intervention based
8	Overton	2/7/2025	6/27/2024	Massachusetts State 911 Department	List of training courses	Record 8 june 27_Approved Co	Emergency Services	Training courses avaliable: 911 dispatchers and call takers managing stress, 911 emergency dispatching: emotional survival for dispatchers and call takers, 911 emergency dispatching: health & wellness issues for dispatch. 911 emergency dispatching: how to avoid dispatcher fatigue, 911: emergency dispatching: how to survive dispatch stress,	None specified	No - course list	No - not intervention based
9	Google	2/7/2025	6/25/2024	East of England Ambulance Service	Our People Strategy 2023-2025	https://www.eastamb.nhs.uk/r	Ambulance	Interventions: publish menopause guidance, launch welbeing champions programme, promote health and wellbeing conversations as a daily rhythmn, aim/aspire to finish on time/commit to rest breaks, increase praise and positive feedback, provide cogitive behavioural therapy / physiotherapy via our occupational health provider with additional sessions if appropriate, purhcase and improve quality and acess to food on our welfare wagons, increase number of trauma risk management and mental health first aiders, establish an internal oversight of occupational health by employment of an inernal occupational health team. In the second and third year: train all staff in mental health awareness, support integrated care system initiatives to reduce handover delays, embed flexible working options.	Outcomes: Better work/life balance, meal breaks and reliable shift patterns are better managed, relief and handover delays have been reduced, access to mental and physical health restoration, safe sharing environment, flexible working opportunities	No - strategy not intervention	No - not intervention based
10	Google	2/7/2025	Mar-23	Nottingham Trent University / The Fire Fighters C	Mapping the Health and Wellbeing across the Firefightir	Record 10 Mapping-the-Health-	Firefighters	Employee Assistance Programmes, peer support (from family and friends and also from co-workers), development of a formal peer support offer which has a robust governance framework, support for transition into retirement and retirement planning, support for personal development and role mainteance, Critical Incident Stress Debriefing (CISD) and Critical Incident Stress Management (CISM), Trauma Risk Management (TRiM). Emphasi on a holistic wellbeing offer	Outcomes measures used: Short Warwick-Edinburgh Mental Wellbeing Scale (SWEMWBS), Depression Anxiety Stress Scales (DASS-21), Ultrecht Work Engagement (UWES-9), Source of Occupational Streaa (SOOS-14), Intention to quit, burnout measure, Impact of Event Scale-Revised (IES-R), Multidimensional Scale of Percieved Social Support, Brief Resilient Coping Scale, Job Satisfactions Scale, Satisfaction with Life Scale (SWLS), Bluelight Awareness of Support	Yes	For discussion - unsure
11	Goolge	2/7/2025	May-21	Association of ambulance chief executives / Public Health England / College of Paramedics / The Ambulance Staff Charity / Health Education England	Prevention of Suicide in the Ambulace Service: What we Know	Record 11 CCS1120525712-00	Ambulance	Mind Blue Light Programme recommendations & cross-government suicide prevention workplan: the first cross government suicide prevention plan (Suicide prevention strategy)		No - no outcomes reported	No - not focussed on researching a specific intervention, and no outcomes reported
12	Google	2/7/2025		MIND	Blue Light Programme - Phae Three Evaluation of 999 Ca	Record 12 blue-light-programm	Ambulance, Fire, Police	Control Room Pledge Refreses, Champions and peer support hints & tips, call handler briefings, managing mental health in the emergency services, workplace wellbeing webinars, adapting existing materials	Post training evaluation forms. Observation of two full day training sessions, two focus group, five in-depth interviews, site visits and observations at three control rooms	Yes	Yes
13	Fire Fighters Chari	2/7/2025		Fire Fighers Charity	HOPE Programme	https://www.firefighterscharity.	Firefighters	Self -directed online wellbeing programme; siz week online self-management programme which helps participants feel more in control and more resilient whilst remaining anonymous. Each week it covers topics like managing stress, smarter goal setting, gratitude, character strengths, physical activity, eating well, coping with fatigue, practicing mindfulness, communication and relationships.		No - no outcomes reported	No - programme link rather than outcomes reported

14	Fire Fighters Chari	2/7/2025		Fire Fighers Charity	Physical Health Support	https://www.firefighterscharity.	Firefighters	Support with recovery from injury, illness or surgery. Support with long term health conditions, lifestyle management and advice, 1:1 digital phsysiotherapy, personalised exercises, residential stays, self help resources, wellbeing apps, execise at home, Reset residential programme		No - no outcomes reported	No - programme link rather than outcomes reported
15	Fire Fighters Chari	2/7/2025	N/A	Fire Fighers Charity	Wellbeing Workshops and Coaching	https://www.firefighterscharity.	Firefighters	Wellbeing Coaching: Sleep, stress, resilience, relaxation and mindfulness, mental health and physical activity, happiness and wellbeing, positive psychology, strong relationships, how connecting with nature is good for our health, improving communication, MSK injury prevention, MSK Injury managaement and rehabilitation, understanding and managing back pain, nutrition and healthy eating, mental health and physical activity, Understanding menopause, wellbeing into retirement, introduction to relaxation and mindfulness, mental health and physical activity, nurtition and healthy eating	Evaluation - completion of online survey, follow up survey 1 and 2 months later.	No - no outcomes reported	No - programme link rather than outcomes reported
16	The Ambulance St	2/7/2025	N/A	The Ambulance Staff Charity	Various resources; health & wellbeing	https://www.theasc.org.uk/ser	Ambulance	Crisis line, mental health (stress, burnout, depression, anxiety, trauma, PTSD, compassion fatigue), bereavement support, financial wellbeing, online wellbeing hub, physical rehabilitation.		No - no outcomes reported	No - programme link rather than outcomes reported
17	Police Care UK	2/7/2025	N/A	Police Care UK	Various informational resources	https://www.policecare.org.uk/	Police Service	Practical, emotional and financial informational resources; PTSD, Anxiety, Bereavement & Death, Alcohol Awareness, Assistance Grants, Counselling		No - no outcomes reported	No - programme link rather than outcomes reported
18	College of Paramedics	2/7/2025	N/A	College of Paramedics	Future workforce mental health & wellbeing project	https://collegeofparamedics.co	Ambulance	Health Education England commissioned the College of Paramedics to undertake a 'Future Workforce Mental Health & Wellbeing Project' in response to the ongoing psychological risks facing the profession, particularly those working in ambulance services. The three interventions identified as a focus for the Project were:<br><br>Development of curriculum guidance regarding personal mental health and well-being for pre-registration Paramedic training.<br>Development of a wellbeing and recovery support tool for use by paramedic students and early career staff in ambulance trusts.<br>Development of a learning and development package for student and preceptorship supervisors.		No - no outcomes reported	No - no outcomes reported
