# Supplementary material for: A Systematic Review of Interventions to Support Health, Wellbeing, and Job Satisfaction for Emergency Call Handlers in the Ambulance, Police, and Fire Services
Source: Stress Health. 2026 Oct 7;42(5):e70233. doi: 10.1002/smi.70233 (PMC13646713; doi:10.1002/smi.70233)
Supplement: Supplementary file 2 [file SMI-42-e70233-s001.docx]

**Appendix A**

Search Strategy

**Appendix A1 – Scopus Search**

(( TITLE-ABS-KEY ( initiative* ) ) OR ( TITLE-ABS-KEY ( support ) ) OR ( TITLE-ABS-KEY ( resource* ) ) OR ( TITLE-ABS-KEY ( program* ) ) OR ( TITLE-ABS-KEY ( "peer support" ) ) OR ( TITLE-ABS-KEY ( training ) ) OR ( TITLE-ABS-KEY ( strateg* ) ) OR ( TITLE-ABS-KEY ( guidance ) ) OR ( TITLE-ABS-KEY ( intervention* ) ) OR ( TITLE-ABS-KEY ( implementation ) )) AND (( TITLE-ABS-KEY ( "mental health" ) ) OR ( TITLE-ABS-KEY ( "physical health" ) ) OR ( TITLE-ABS-KEY ( sickness ) ) OR ( TITLE-ABS-KEY ( illness ) ) OR ( TITLE-ABS-KEY ( exercise ) ) OR ( TITLE-ABS-KEY ( drinking ) ) OR ( TITLE-ABS-KEY ( alcohol ) ) OR ( TITLE-ABS-KEY ( smoking ) ) OR ( TITLE-ABS-KEY ( sleep ) ) OR ( TITLE-ABS-KEY ( food ) ) OR ( TITLE-ABS-KEY ( "healthy eating" ) ) OR ( TITLE-ABS-KEY ( wellbeing ) ) OR ( TITLE-ABS-KEY ( fatigue ) ) OR ( TITLE-ABS-KEY ( stress ) ) OR ( TITLE-ABS-KEY ( mood ) ) OR ( TITLE-ABS-KEY ( burnout ) ) OR ( TITLE-ABS-KEY ( exhaustion ) ) OR ( TITLE-ABS-KEY ( happiness ) ) OR ( TITLE-ABS-KEY ( "job satisfaction" ) ) OR ( TITLE-ABS-KEY ( workforce ) ) OR ( TITLE-ABS-KEY ( "staff retention" ) ) OR ( TITLE-ABS-KEY ( absenteeism ) )) AND (( TITLE-ABS-KEY ( "emergency call handler*" ) ) OR ( TITLE-ABS-KEY ( clinician* ) ) OR ( TITLE-ABS-KEY ( "call taker*" ) ) OR ( TITLE-ABS-KEY ( dispatch* ) ) OR ( TITLE-ABS-KEY ( "call handler*" ) ) OR ( TITLE-ABS-KEY ( "999" ) ) OR ( TITLE-ABS-KEY ( "911" ) ) OR ( TITLE-ABS-KEY ( "call centre" ) ) OR ( TITLE-ABS-KEY ( "dispatch officer" ) ) OR ( TITLE-ABS-KEY ( "support staff" ) )) AND (( TITLE-ABS-KEY ( ambulance ) ) OR ( TITLE-ABS-KEY ( police ) ) OR ( TITLE-ABS-KEY ( fire ) ))

**Appendix A2 – EBSCO Search**

**Appendix A3 – EMBASE Search**

**Appendix A4 – Summary of Grey Literature Searches**

Search Strategy

“initiative*” OR “support” OR “resource*” OR “program*” OR "peer support" OR “training” OR “strateg*” OR “guidance” OR “intervention*” OR “implementation” AND "mental health" OR "physical health" OR “sickness” OR “illness” OR “exercise” OR “drinking” OR “alcohol” OR “smoking” OR “sleep” OR “food” OR "healthy eating" OR “wellbeing” OR “fatigue” OR “stress” OR “mood” OR “burnout” OR “exhaustion” OR “happiness” OR "job satisfaction" OR “workforce” OR "staff retention" OR “absenteeism” AND "emergency call handler*" OR “clinician*” OR "call taker*" OR “dispatch*” OR "call handler*" OR "999" OR "911" OR "call centre" OR "dispatch officer" OR "support staff" AND “ambulance” OR “police” OR “fire”
