# Supplementary material for: A Systematic Review of Interventions to Support Health, Wellbeing, and Job Satisfaction for Emergency Call Handlers in the Ambulance, Police, and Fire Services
Source: Stress Health. 2026 Oct 7;42(5):e70233. doi: 10.1002/smi.70233 (PMC13646713; doi:10.1002/smi.70233)
Supplement: Supplementary file 3 [file SMI-42-e70233-s003.docx]

**Appendix B**

Data Extraction Table (Blank)
